# Unraveling the Lignin Structural Variation in Different Bamboo Species

**DOI:** 10.3390/ijms241210304

**Published:** 2023-06-18

**Authors:** Ling-Ping Xiao, Yi-Hui Lv, Yue-Qin Yang, Shuang-Lin Zou, Zheng-Jun Shi, Run-Cang Sun

**Affiliations:** 1 Liaoning Key Lab of Lignocellulose Chemistry and BioMaterials, Liaoning Collaborative Innovation Center for Lignocellulosic Biorefinery, College of Light Industry and Chemical Engineering, Dalian Polytechnic University, Dalian 116034, China; lyhui723@163.com (Y.-H.L.); y2276735283@163.com (Y.-Q.Y.); shuanglinzz@163.com (S.-L.Z.); 2 Key Laboratory for Forest Resources Conservation and Utilization in the Southwest Mountains of China, Ministry of Education, Southwest Forestry University, Kunming 650224, China; shizhengjun1979@swfu.edu.cn

**Keywords:** bamboo, lignin, structural characterization, hydrogenolysis, phenolic monomers

## Abstract

The structure of cellulolytic enzyme lignin (CEL) prepared from three bamboo species (*Neosinocalamus affinis*, *Bambusa lapidea*, and *Dendrocalamus brandisii*) has been characterized by different analytical methods. The chemical composition analysis revealed a higher lignin content, up to 32.6% of *B. lapidea* as compared to that of *N. affinis* (20.7%) and *D. brandisii* (23.8%). The results indicated that bamboo lignin was a *p*-hydroxyphenyl-guaiacyl-syringyl (H-G-S) lignin associated with *p*-coumarates and ferulates. Advanced NMR analyses displayed that the isolated CELs were extensively acylated at the γ-carbon of the lignin side chain (with either acetate and/or *p*-coumarate groups). Moreover, a predominance of S over G lignin moieties was found in CELs of *N. affinis* and *B. lapidea*, with the lowest S/G ratio observed in *D. brandisii* lignin. Catalytic hydrogenolysis of lignin demonstrated that 4-propyl-substituted syringol/guaiacol and propanol guaiacol/syringol derived from β-*O*-4′ moieties, and methyl coumarate/ferulate derived from hydroxycinnamic units were identified as the six major monomeric products. We anticipate that the insights of this work could shed light on the sufficient understanding of lignin, which could open a new avenue to facilitate the efficient utilization of bamboo.

## 1. Introduction

As the cell wall component of terrestrial plants, lignin is widely distributed in nature, and its content is second only to (hemi)cellulose [1,2]. Lignin contains a complex structure of the phenolic polymer, which is especially suitable for the production of aromatic chemicals, and its components vary with different plant materials [3,4,5,6,7,8]. However, most biorefinery schemes focus on the use of easy-to-use compositions, while lignin is relatively underutilized [9,10,11]. For example, about one million t/y of lignosulfonate accounted for only 2% of the total production for commercialization [12,13]. Catalytic conversion of lignin for the production of small molecule chemicals or fuels has received much attention because of its high yield and widespread availability [14]. Nevertheless, it is still a significant challenge for lignin valorization in biorefinery owing to its complexity and heterogeneity of the internal macromolecular structure [15,16]. To solve the above-mentioned challenges, it is necessary to gain a broad understanding of the “main” structural characteristics of lignin to provide a theoretical basis for biomass upgrading, pulping, and biorefinery [14,17].

The content and composition of lignin vary with wood type, cell type and single cell wall layer, and environmental conditions [18]. In recent years, herbaceous plants, such as bamboo and hemp, have gradually attracted extensive attention due to their short growth cycle and mild growth conditions. As typical of fast-growing plants, most of the herbaceous plants contain relatively low lignin contents but with high levels of hydroxycinnamic acid, i.e., *p*-coumaric acid (*p*CA) and ferulic acid (FA) [19,20,21,22] Generally, cellulolytic enzyme lignin (CEL), which is obtained by extraction of the residue from cellulase treated ball-milled materials with 96% dioxane solvent, is more representative of the total lignin in lignocellulosic biomass [23,24]. The structural characterization of lignin macromolecules isolated from different hardwood and bamboo species [17,18,25] using advanced nuclear magnetic resonance (NMR) technologies, inducing ^13^C, ^31^P, and 2D HSQC, could facilitate the development of efficient utilization strategies to meet current biorefinery toward a circular economy [26].

Over the years, more and more scientific researchers used precious metals (Pd [27,28,29,30,31,32,33], Ru [5,27,34,35,36,37,38], and Pt [39,40,41]) or non-precious metals (Ni [42,43,44,45,46], Fe [47,48], Mo [49,50], and Cu [51,52]) for the catalytic transformation of lignin into chemicals and fuel products. For example, Luterbacher and co-workers [34] reported that adding formaldehyde during biomass pretreatment produced a soluble lignin fraction that could be converted into guaiacyl and syringyl monomers at near theoretical yields (47 mole% of Klason lignin for beech and 78 mole% for a high-syringyl transgenic poplar) during subsequent hydrogenolysis using a Ru/C catalyst. We have recently developed ruthenium nanoparticles (NPs) anchored on defective nitrogen-doped carbon (Ru@NC) via facile pyrolysis of a mixture of ruthenium trichloride and urea with carbon support [53]. Experimental insights indicated that the highly distributed Ru-NPs, constituted by N-enriched graphene shells, have been established as an excellent catalyst for the selective hydrodeoxygenation of lignin and furan derivatives toward biofuel upgrade [53]. As a continuation of our ongoing interest in effectively developing reductive catalytic depolymerization of lignin, we envision that this catalyst exhibits efficient performance for the hydrogenolysis of lignins and β-*O*-4′ model compounds through the scission of C–O bonds.

In this study, to better unravel the lignin structural variation in three bamboo species (*Neosinocalamus affinis*, *Bambusa lapidea*, and *Dendrocalamus brandisii*), which are widely grown in southwest China, typical CEL preparations were successively isolated from different bamboo species. The composition and structures of the obtained CEL fractions were comprehensively investigated. In order to obtain further insights into their structures, the CEL samples were also analyzed by catalytic hydrogenolysis. The results of this work are important not only for providing new insights into the bamboo lignin characteristics but also for the industrial processing of bamboo for pulp, chemical, or biofuel production.

## 2. Results and Discussion

### 2.1. Composition Analysis of Bamboo

The contents of main constituents (i.e., Klason lignin, acid-soluble lignin, glucan, xylan, arabinan, galactan, rhamnan, mannan, glucuronic acid, galacturonic acid, and ash) in the selected three bamboo species (*N. affinis*, *B. lapidea*, and *D. brandisii*) are summarized in Table 1. Quantitative measurement of lignin is an important aspect of the study of lignin structure [54]. It was observed that the total lignin content (Klason lignin plus acid-soluble lignin) of *B. lapidea* amounted to 32.6%, which was significantly higher than those of *N. affinis* (20.8%) and *D. brandisii* (23.8%). This result was consistent with previously reported *Phyllostachys pubescens* (26.1~28.2%) [12,55,56,57] and *Dendrocalamus sinicus* (28.6%) [58]. After a general analysis of the chemical composition of the three bamboo species, the variations in the isolated CEL preparations were systematically analyzed, especially with the advanced NMR technologies (^13^C, ^31^P, and 2D HSQC).

### 2.2. FT-IR Spectroscopy

**Figure 1:**
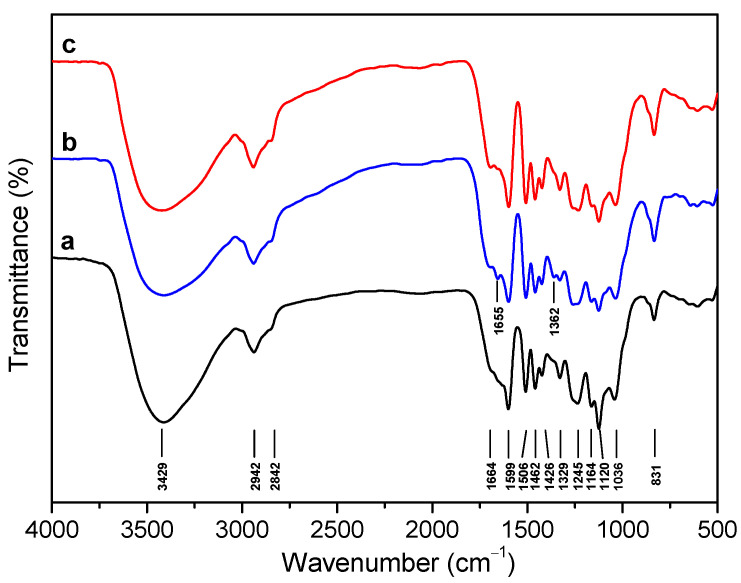
FT-IR spectra of (a) CEL_N_, (b) CEL_B_, and (c) CEL_D_. CEL_N_, CEL_B_, and CEL_D_ were isolated from *N. affinis*, *B. lapidea*, and *D. brandisii*, respectively.

Figure 1 shows the FT-IR spectra of lignin extracted from three species of bamboo, which can identify the characteristic functional groups [33,34,35]. Obviously, the broad absorption band at 3429 cm^−1^ is associated with the OH stretching vibration. The bands at 1664 and 1655 cm^−1^ indicate the unconjugated carbonyl of the keto group and the carbonyl stretch of the conjugated carbonate, respectively. The peaks at 1599, 1511, and 1426 cm^−1^ correspond to aromatic backbone vibrations and C-H deformations, and the methoxy group at 1460 cm^−1^ is the asymmetric C-H vibration. The analysis of the spectra of the three lignins demonstrated that the aromatic skeleton of the lignin structure was kept well during the isolation process [12]. Syringyl and condensed guaiacyl absorb the band at 1239 cm^−1^, and the fused G-unit with the C–O stretching at 1253 cm^−1^ [58,59,60]. The 1125 and 834 cm^−1^ peaks dominate as unambiguous signals for HGS lignin, which proves that the three bamboo lignins exhibit the pattern of typical grass lignin.

### 2.3. Molecular Weight Distribution and Thermal Stability

**Figure 2:**
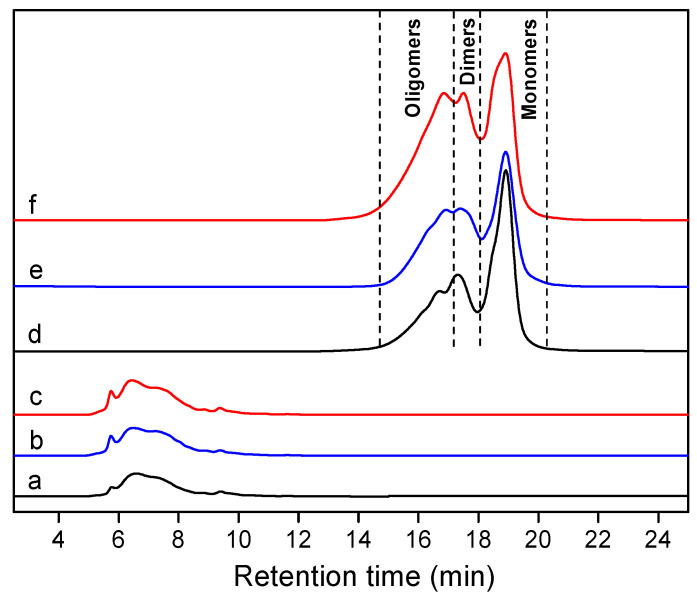
GPC of (a) CEL_N_, (b) CEL_B_, (c) CEL_D_ and lignin oil obtained from the catalytic hydrogenolysis of (d) CEL_N_, (e) CEL_B_, (f) CEL_D_ over a Ru@NC catalyst.

The molecular weight distribution of the CEL preparations after acetylation was determined by gel permeation chromatography (GPC). It was observed that CEL_N_ displayed the lowest weight average molecular weight (M_w_) of 8.13 kDa in comparison with those of CEL_B_ (9.08 kDa) and CEL_D_ (9.55 kDa) in Figure 2. However, all these data were comparable with previously reported bamboo CELs [61,62,63]. Moreover, the polydispersity index (M_w_/M_n_, Ð) of the three CELs was found to be relatively narrow (Ð < 3.0) and with no significant differences (Table 2).

**Figure 3:**
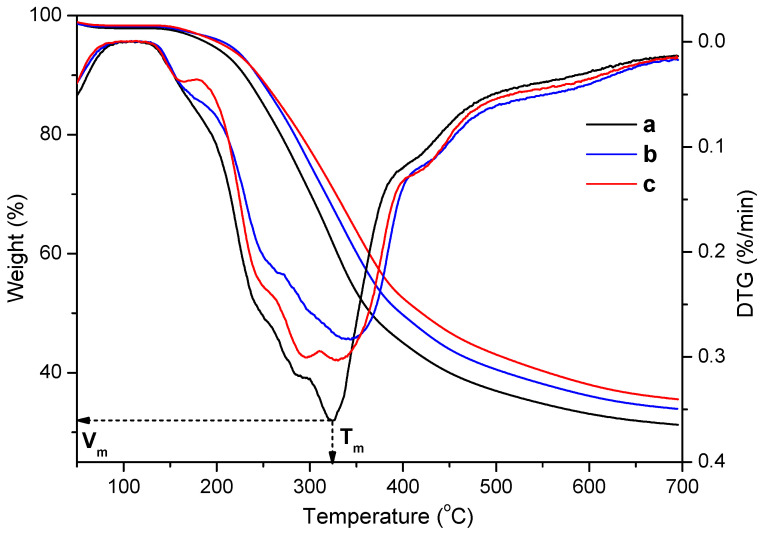
TG and DTG curves of (a) CEL_N_, (b) CEL_B_, and (c) CEL_D_.

As depicted in Figure 3, the percentages of residues at different stages and the corresponding temperatures (T_m_) at the maximum rate of quality loss were obtained from the TG and DTG curves of the three bamboo CELs. Among them, CEL_N_ showed the lowest residual amount, whereas CEL_B_ and CEL_D_ were in an incremental residual amount, indicating that CEL_N_ was lower in thermal stability. Moreover, a three-stage weight loss process was observed in the TG curve. The first stage was a slight weight loss due to the volatilization of bound water and loss of the residue extracts at 50~200 °C. The pyrolysis rate was high in the second stage at 200~500 °C, which was due to the pyrolysis of carbohydrates and lignin. The curve in the third stage gradually flattened, which was mainly attributed to the degradation of tar and coke. As discussed above, lignin was easier to convert into char due to its highly agglomerated properties [64,65]. The cleavage of the β-*O*-4′ bonds mainly occurred at 250~350 °C [66]. By analyzing the DTG curves, CEL_N_ exhibited the representative lowest decomposition peak, which might be related to the content of β-*O*-4′ linkages in it. Therefore, it could be concluded that the thermal stabilities of CEL_B_ and CEL_D_ were relatively higher than that of CEL_N_.

### 2.4. NMR Characterization

**Figure 4:**
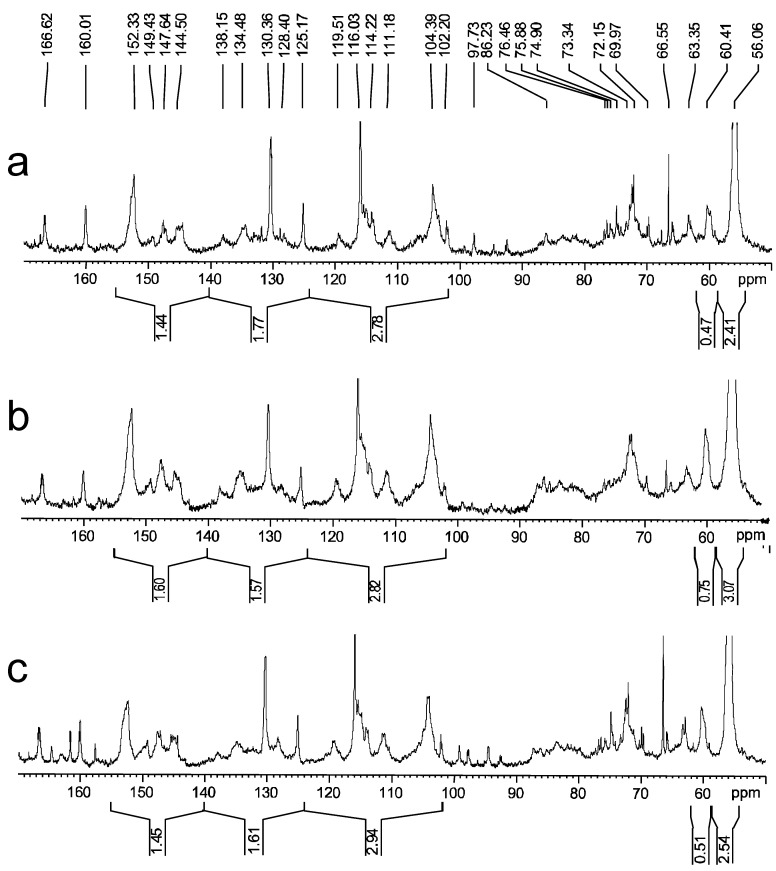
Quantitative ^13^C NMR spectra of (**a**) CEL_N_, (**b**) CEL_B_, and (**c**) CEL_D_.

The ^13^C NMR spectroscopy provides valid evidence for the analysis of the chemical structure of bamboo lignin [41,42,43]. As shown in Figure 4, the peaks of S-type were identified by signals at 151.1–155.1 ppm (C-3/C-5 etherified and condensed), 146.6–148.7 ppm (C-3/C-5 non-etherified), 138.1 ppm (C-4 etherified), 133.0–136.6 ppm (C-1), and 102.5–106.5 ppm (C-2/C-6). The G-units give signals at 148.7–151.0 ppm (C-3 etherified and condensed), 146.6–148.7 ppm (C-4 etherified), 144.0–146.6 ppm (C-3/C-4 non-etherified), 133.0–136.6 ppm (C-1), 119.5 ppm (C-6), 113.1–118.0 ppm (C-5), and 110.0–113.1 ppm (C-2). The H units were detected at 128.1 ppm (C-2/C-6), and 113.1–118.0 ppm (C-3/C-5). Moreover, the *p*CA was evidenced by five signals at 166.7, 160.1, 130.5, 125.3, and 113.1–118 ppm, which originated from C-9, C-4, C-2/C-6, C-3/C-5, and C-β in the structures, respectively. Additionally, we assigned the above spectral regions to functional groups and quantified them by integration (Table S1). The analysis results of quantitative ^13^C NMR spectroscopy further revealed that the bamboo CEL was a typical HGS-type lignin, which was in good accordance with the aforementioned results of FT-IR.

**Figure 5:**
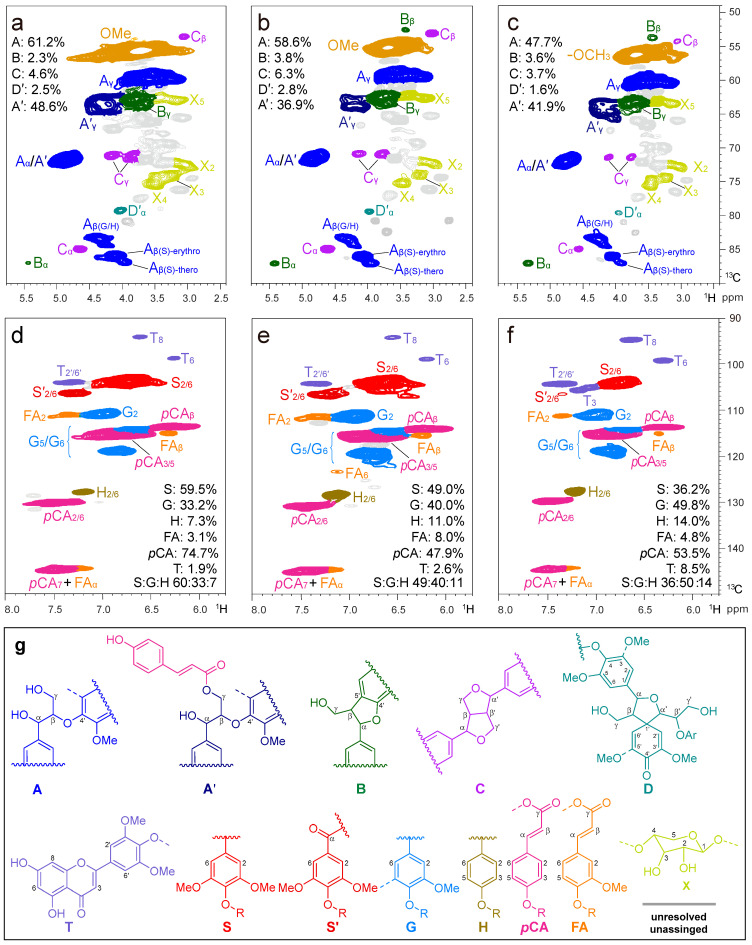
2D HSQC NMR spectra of the prepared (**a**,**d**) CEL_N_, (**b**,**e**) CEL_B_, and (**c**,**f**) CEL_D_ isolated from ball-milled bamboo. The colors of the contours correspond to the structures drawn. (**g**) The main structures and lignin-derived monomers found are as follows: (A) β-*O*-4′ alkyl-aryl ether; (A′) γ-OH with *p*-coumaroylated β-*O*-4′ alkyl-aryl ethers; (B) phenylcoumarans; (C) resinols; (D) spirodienones; (T) tricin; (S) syringyl units; (S′) oxidized syringyl units bearing a carbonyl at C_α_; (G) guaiacyl units; (H) *p*-hydroxyphenyl unites; (*p*CA) *p*-coumarates; and (FA) ferulates.

2D HSQC NMR spectrum has been reported to be capable of providing important structural information about the complex polymer substructures bonded between lignin units and S/G ratio. The aliphatic (δ_C_/δ_H_ 10–45/0–3.5 ppm), aliphatic-oxygenated (δ_C_/δ_H_ 49–90/2.4–5.8 ppm), and aromatic (δ_C_/δ_H_ 90–150/5.7–8.0 ppm) regions of the 2D HSQC NMR of CEL_N_ are illustrated in Figure 5. The assignments of main lignin cross-signals in the HSQC spectra were assigned according to previously reported literature [12,58,62,67,68,69,70,71], which are listed in Table S2, and the main substructures are depicted in Figure 5.

The aliphatic-oxygenated region of the spectra (Figure 5b) provided information about the different interunit linkages present in the lignin. In this region, correlation peaks from methoxyls and side chains in β-*O*-4′ substructures (A, 61.2%) were the most prominent in the HSQC spectra of the isolated CEL_N_. Other substructures were also visible in the HSQC spectrum of the CEL_N_, including signals for phenylcoumarans (B, 2.3%) and resinols (C, 4.6%). Notably, a large predominance of β-*O*-4′ linkages (up to 48.6% of all linkages) was observed at δ_C_/δ_H_ 63.5/4.21, which was assigned to the γ-acylated lignin units A′. This indicated that the structure of native lignin was highly remarkable, being extensively acylated (acetylated and/or *p*-coumaroylated) in bamboo [69]. The main cross-signals from S, G, and H units are visible in the aromatic region of the HSQC spectra (Figure 5c), which correspond to the benzenic rings of lignin units. The prominent signal at δ_C_/δ_H_ 103.9/6.58 ppm was associated with C_2,6_-H_2,6_ in S-type units. The peaks at δ_C_/δ_H_ 110.9/6.93, 114.4/6.69, and 118.9/6.80 ppm were assigned to C_2_-H_2_, C_5_-H_5_, and C_6_-H_6_ of the G-type unit, respectively. The C_2,6_-H_2,6_ of the H-type hydroxyphenyl building block appeared at δ_C_/δ_H_ 127.8/7.28 ppm. In addition, the *p*CA and FA units were detected in the HSQC spectra. The series of signals at δ_C_/δ_H_ 130.0/7.35, 115.8/6.81, and 113.8/6.20 ppm were associated with C_2,6_-H_2,6_, C_3,5_-H_3,5_, and C_β_-H_β_ in *p*CA, respectively. The crossover signals of C_2_-H_2_ and C_β_-H_β_ in FA appeared at δ_C_/δ_H_ 110.9/7.25 and 115.3/6.29 ppm. In addition, the HSQC spectra showed signals of C_8_-H_8_ and C_6_-H_6_ at δ_C_/δ_H_ 94.3/6.62 and 98.9/6.15 ppm, respectively, which were attributed to the grass-specific tricin (T) lignin units [72,73].

To further investigate the differences in the chemical structures of the three bamboo lignin species, quantitative ^31^P NMR technique was employed (Figure S4), and the quantitative data on the distribution of different OH groups in the CELs are listed in Table S3 [57]. The results of phenolic OH content revealed no significant differences among the three CELs. However, the highest content of aliphatic hydroxyl groups was detected in CEL_B_, and the lowest content of total hydroxyl groups in CEL_N_.

### 2.5. Catalytic Hydrogenolysis of Lignin

Catalytic hydrogenolysis was developed to produce aromatic products and phenolic moieties from lignin. Typically, alkyl aryl ether linkages in the lignin biomacromolecules are cleaved during this process. Moreover, secondary (benzylic) alcohols are removed, and aliphatic double bonds are reduced, which provides more additional information regarding the characteristics of the lignin side chain. On treatment of bamboo CEL_N_ with 10 wt% of Ru@NC at 240 °C and 3 MPa of H_2_ in MeOH for 4 h in a Parr autoclave, a brown soluble oily product was obtained after extraction with CH_2_Cl_2_ (Figure S1). This catalytic hydrogenolysis process afforded a monophenol yield of 26.6 wt% (Table 1), and the detailed monomer distribution is depicted in Figure S5. Both syringyl- and guaiacyl-derived phenols were detected with an S/G ratio of 2.2, slightly higher than that of S/G monomer composition in the original lignin (1.8). Among the monomers, 4-propyl-substituted syringol (S1, 12.2 wt%) and guaiacol (G1, 4.1 wt%) were identified as the two major products, corresponding to 61.9 mol% selectivity of total monomers. Small quantities of 4-*n*-propanol guaiacol/syringol (G2/S2, 5.1 wt%) were also detected (Table 3). As a typical herbaceous species, bamboo lignin features hydroxycinnamic acid, which is bonded with α-OH or γ-OH of β-*O*-4′ moieties through ester or ether linkages [74,75]. Accordingly, two specific phenolic monomers (3, 4.1 wt%; 4, 1.1 wt%) were also generated from the *p*CA and FA units, which amounted to 20.9 mol% selectivity of total monomers. Moreover, the analysis of the oily product (CELO_N_) and by GPC revealed a significant decrease in molecular weight (M_w_ 0.66 kDa) relative to the initial CEL_N_ (M_w_ 8.13 kDa) (Table 2). These results demonstrated the successful scission of most C–O bonds under such a catalytic condition.

**Figure 6:**
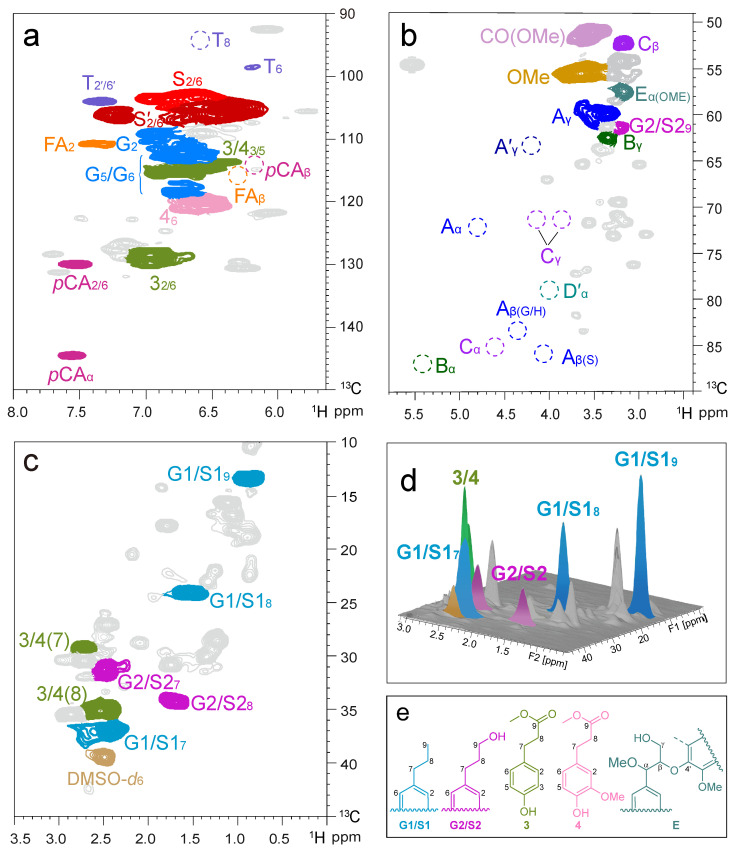
2D HSQC NMR spectra (in DMSO-*d*_6_) of the lignin oily product (**a**–**c**) obtained from the Ru@NC-catalytic hydrogenolysis of CEL_N_ (in DMSO-*d*_6_); (**d**) three-dimensional version of the 2D HSQC end-chain region; (**e**) detected lignin monomers. Reaction conditions: CEL_N_ (50 mg), Ru@NC catalyst (5 mg, 10 wt%), MeOH (10 mL), 240 °C, H_2_ (3 MPa at 25 °C, 12 MPa at 240 °C), and 4 h.

The as-obtained oily product was further characterized by 2D HSQC NMR spectroscopy (Figure 6). All signals of monomeric phenols were assigned based on the comparison with authentic samples. Notably, no detectable signals for lignin linkages remained in Figure 6b, such as β-aryl ether (β-*O*-4′, A), phenylcoumaran (β-5, B), and resinol (β–β, C), indicating efficient depolymerization of lignin over the Ru@NC catalyst. The cross peaks at δ_C_/δ_H_ 13.5/0.85, 24.5/1.53, and 36.6/2.48 ppm ascribed to the propyl end chains in G1/S1 could be easily observed (Figure 6c). A family of cross peaks located at δ_C_/δ_H_ 31.8/2.51, 34.6/1.71, and 61.3/3.25 emerged, relating to the propyl end chain in G2/S2. In addition, signal at δ_C_/δ_H_ 51.0/3.58 was attributed to the ester group (CO_2_Me) in the product (Figure 6b). In the aliphatic region, the signal peaks of the C_7_, C_8_, and C_9_ belonging to G- and S-products were found, while the signal peaks of 3 and 4 were mainly found in the aromatic region (Figure 6a,c). The characteristic of the monomers was further verified by the addition of the 3D version in Figure 6d.

We further evaluated the catalytic hydrogenolysis performance of the other two bamboo lignin species over the Ru@NC catalyst (Figures S2 and S3). Notably, CEL_B_ afforded a relatively higher yield of phenolic monomer (26.5 wt%) than that of CEL_D_ (16.5 wt%), which was due to the high proportion of β-*O*-4′ linkages in CEL_B_ [15,71]. As expected, both CEL_B_ and CEL_D_ gave G1 and S1 as the dominant products (60.5~64.6% selectivity). Moreover, the 2D HSQC NMR spectroscopies of lignin oils from those two lignin preparations indicated that there were no β-*O*-4′ structures existing, illustrating the fullest cleavage of C–O linkages, which was in good accordance with the GPC analysis in Table 2 and Figure 2.

### 2.6. Catalytic Hydrogenolysis of Lignin β-O-4′ Model Compounds

**Figure 7:**
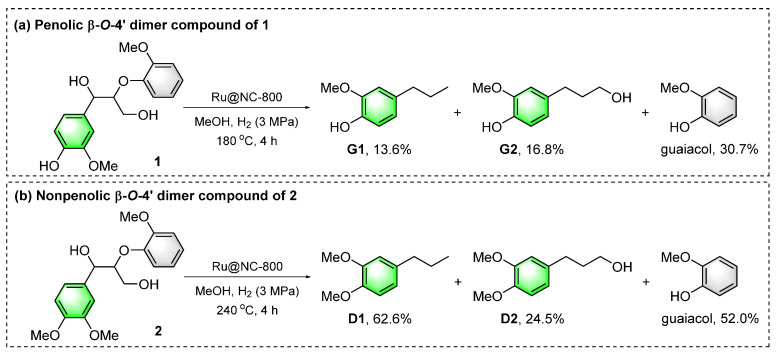
Catalytic hydrogenolysis of the lignin β-*O*-4′ model compounds over a Ru@NC catalyst.

To further understand the pathway of lignin during the Ru@NC-catalyzed hydrogenolysis reactions, the typical lignin β-*O*-4′ model compounds were tested (Figure 7) [76,77]. Catalytic hydrogenolysis of phenolic β-*O*-4′ dimer 1 offered G1 (13.6%) and G2 (16.8%) as the major phenolic monomer products (reaction a, Figure S6). Nonphenolic β-*O*-4′ dimer 2 could efficiently afford 4-propylveratrole (D1) and benzenepropanol (D2) in 62.6% and 24.5% yields, respectively (reaction b, Figure S7). All the product distributions were similar to those of the Ru@NC-catalyzed hydrogenolysis of CEL samples. These results further confirmed that Ru@NC displayed excellent activity for the hydrogenation of C=C/C=O bonds and hydrogenolysis of C–O bonds in β-*O*-4′ moieties.

### 2.7. Elemental Composition of Bamboo, Lignin, and Lignin Oil

**Figure 8:**
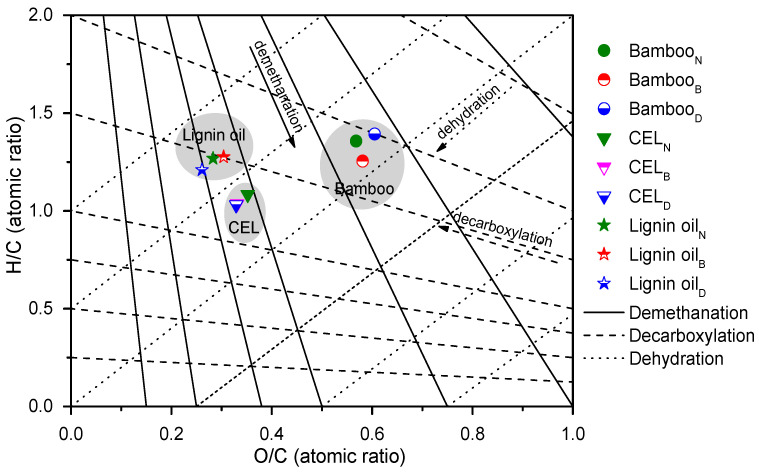
Atomic H/C versus O/C ratios (van Krevelen diagram) of bamboo, bamboo CELs, and lignin oil obtained from the Ru@NC-catalytic hydrogenolysis of CELs. The lines represent demethanation, dehydration, and decarboxylation pathways. Bamboo_N_, Bamboo_B_, and Bamboo_D_ refer to *N. affinis*, *B. lapidea*, and *D. brandisii*, respectively.

The elemental compositions of the three bamboo species, CELs, and the corresponding lignin oily products were investigated, which is summarized in Table S4 and depicted in Figure 8 as the van Krevelen diagram [32,78]. The solid, dashed, and dotted lines in the diagram represent the processes of demethanation, decarboxylation, and dehydration reactions, respectively. Notably, the H/C ratio of Bamboo_B_ was slightly lower than that of the other two bamboo samples (Bamboo_N_ and Bamboo_D_) because of the lower content of carbohydrates, which was consistent with the compositional analysis results in Table 1. It was found that the average H/C molar ratios of lignin oil were significantly higher than those of CELs. This was reasonable as (hemi)celluloses were removed in the preparation of CEL samples, which was further verified by the O/C ratios between bamboo and CEL samples. Moreover, it was found that the average O/C molar ratio of lignin oil products (0.26~0.30) was close to those of CEL samples (0.33~0.35) but much lower than those of bamboo materials (0.57~0.61). The results of reduced oxygen content but increased hydrogen content further indicated the efficient C–O bond scission in the catalytic hydrogenolysis of lignin, which led to deoxygenation and decarboxylation process.

## 3. Materials and Methods

### 3.1. Materials

Three bamboo species (*N. affinis*, *B. lapidea*, and *D. brandisii*) were harvested from Yunnan Province, China. Methanol (MeOH), dichloromethane (DCM), and tetrahydrofuran (THF) were purchased from Energy Chemical (Shanghai, China). The commercial cellulase was kindly provided by Novozymes (Beijing, China) Biotechnology Co., Ltd. Lignin model samples for catalytic degradation were synthesized independently. Dimeric lignin model compounds were prepared following previously reported procedures with modifications [49,52,79]. The Ru@NC catalyst used in this work was prepared and described in detail as previously described [53].

### 3.2. Preparation of Cellulolytic Enzyme Lignin

The bamboo raw materials were smashed into sawdust (40~60 mesh), dried in an oven at 60 °C, and then extracted with ethanol/toluene (1:2, *v*/*v*) using a Soxhlet extractor for 10 h. The preground and extracted bamboo samples were then planetary ball milled (Fritsch GmbH, Idar-Oberstein, Germany) at 400 rpm for 4 h with zirconium dioxide (ZrO_2_) vessels containing ZrO_2_ ball bearings (10 mm × 30). One cycle of the ball-milling condition consists of a 10 min milling and a 10 min cooling cycle. Subsequently, the ball-milled samples were subjected to digestion (72 h × 2) to obtain enzyme lignin samples by cellulose at 50 °C in NaOAc buffer (pH 4.8). After that, the solid residue was obtained after centrifugation (5000 rpm for 5 min), washing three times with deionized water, lyophilization, and finally ladled as CEL.

### 3.3. Chemical Components Analysis

The structural carbohydrates and lignin, as well as ash in the dewaxed bamboo sawdust, were determined according to the standard analytical procedures (NREL/TP-510-42618 and NREL/TP-510-42622) [80,81].

### 3.4. Catalytic Hydrogenolysis of Lignin or Lignin Model Compounds

Typically, CEL (50 mg) or lignin model compounds (15 mg), Ru@NC (5 mg), and MeOH (10 mL) were charged into an autoclave (50 mL, Parr Instrument Company, Moline, IL, USA), which was then flushed with N_2_ for three times and pressurized with 3 MPa H_2_ at room temperature. Afterwards, the mixture was stirred at 800 rpm and heated to the desired temperature. After the reaction, the autoclave was cooled and depressurized carefully. The reaction mixture was filtered through a nylon 66 membrane filter (0.22 μm), and the insoluble fraction was washed with DCM. Lignin oily product was obtained after removing DCM under a vacuum condition. An external standard (1,3,5-trimethoxybenzene) was added to the lignin oily solution in DCM.

### 3.5. Characterizations

Gas chromatography-mass spectrometry (GC-MS) and GC analysis were performed for qualitative and quantitative analysis of the aromatic monomers, respectively, as described previously [15,49,52,71].Briefly, GC-MC analyses of the lignin oily product were carried out on a Shimadzu GCMS-QP2010SE equipped with an HP-5 MS (30 m × 250 μm × 0.25 μm, Agilent, Santa Clara, CA, USA) capillary column and an MS detector. GC analyses were conducted with a Shimadzu GC-2010 equipped with an HP-5 column (30 m × 250 μm × 0.25 μm, Agilent) and a flame ionization detector. The monomeric yield obtained from lignins and β-*O*-4′ model compounds were calculated using Equations (1) and (2), respectively:

(1) $$\mathrm{Monomer}\;\mathrm{yield}\;(\mathrm{wt}\%)=\frac{w(monomer)}{w(initiallignin)}\times 100\%$$

(2) $$\mathrm{Monomer}\;\mathrm{yield}\;(\mathrm{wt}\%)=\frac{Mole(monomer)}{Mole(ligninmimics)}\times 100\%$$

Advanced NMR technologies, including ^13^C, ^31^P, and 2D HSQC, were used, and analysis of lignin or lignin oily products was performed on a Bruker Ascend-400 MHz spectrometer instrument (Bruker, Hanau, Germany) [52,71]. The molecular weights of lignin and lignin oil were determined by GPC as described previously [52,71].

## 4. Conclusions

In summary, the structure characterization of CEL isolated from three bamboo species (*N. affinis*, *B. lapidea*, and *D. brandisii*) was investigated. The chemical composition analysis revealed a higher lignin content, up to 32.6% of *B. lapidea* in comparison with that of *N. affinis* (20.7%) and *D. brandisii* (23.8%). The results showed that bamboo lignin was an H-G-S lignin associated with *p*-coumarates and ferulates, indicating typical characteristics of herbaceous lignin. Moreover, advanced NMR analyses displayed that CEL was extensively acylated at the γ-carbon of the lignin side chain (with either acetate and/or *p*-coumarate groups). A predominance of S over G lignin moieties was found in CELs of *N. affinis* and *B. lapidea*, with the lowest S/G ratio observed in *D. brandisii* lignin. The catalytic hydrogenolysis of lignin provided deep information on the well-defined low-molecular-weight phenols, giving evidence on the relative abundances of the various C–O bonds and the type of units involved in each of the linkage types. Six major monophenols, e.g., 4-propyl-substituted syringol/guaiacol and propanol guaiacol/syringol derived from β-*O*-4′moieties, and methyl coumarate/ferulate derived from hydroxycinnamic units, were generated in the range of 16.5~26.6 wt% yields. A sufficient understanding of the structural characteristics of lignin macromolecules in bamboo will facilitate the subsequent utilization of lignocellulosic biomass in an integrated biorefinery.

## Figures and Tables

**Table 1:** Abundances (%) of the main constituents of three bamboo species ^a^.

Constituents	Bamboo Species		
	*N. affinis*	*B. lapidea*	*D. brandisii*
Total Lignin	20.78	32.57	23.78
Klason lignin	18.29	28.73	23.06
Acid-soluble lignin	2.49	3.84	0.72
Cellulose (as glucan)	50.82	45.93	53.19
Hemicellulosic sugars	25.84	18.72	22.17
Xylan	22.94	16.19	20.47
Arabinan	1.13	0.85	0.75
Galactan	0.51	0.43	0.28
Rhamnan	0.02	0.20	0.15
Mannan	0.37	0.28	0.30
Glucuronic acid	0.87	0.66	0.17
Galacturonic acid	ND ^b^	0.11	0.05
Ash	2.13	1.65	0.67

^a^ The structural carbohydrates, lignin, and ash in bamboo sawdust were determined according to the laboratory analytical procedures (LAPs) of the National Renewable Energy Laboratory. ^b^ ND, not detectable.

**Table 2:** Weight-average (M_w_), number-average (M_n_) molecular weights, and the polydispersity index (M_w_/M_n_, Ð) of bamboo CELs and the corresponding lignin oil products.

	Lignin Samples			Lignin Oily Products		
	CEL_N_	CEL_B_	CEL_D_	CELO_N_	CELO_B_	CELO_D_
M_w_ (kDa)	8.13	9.08	9.55	0.66	0.70	0.71
M_n_ (kDa)	3.36	3.30	3.36	0.55	0.59	0.55
Ð	2.4	2.4	2.8	1.2	1.2	1.3

**Table 3:** Catalytic hydrogenolysis of bamboo CEL over a Ru@NC catalyst ^a^.

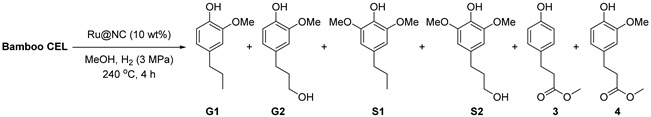												
Lignin Samples	β-*O*-4′ ^b^	S/G		Phenolic Monomer Yield (wt%) ^d^							Selectivity (G1/S1) (mol%) ^e^	Selectivity (3 and 4) (mol%) ^e^
		Before ^b^	After ^c^	G1	G2	S1	S2	3	4	Total		
CEL_N_	61.2	1.8	3.0	4.1	1.8	12.2	3.3	4.1	1.1	26.6	61.9	20.0
CEL_B_	58.6	1.2	2.5	3.2	1.3	12.8	2.8	5.5	0.9	26.5	60.5	24.9
CEL_D_	47.7	0.7	2.5	2.9	0.3	7.6	1.0	2.7	2.0	16.5	64.6	28.1

^a^ Reaction conditions: CEL (50 mg), Ru@NC catalyst (50 mg), MeOH (10 mL), H_2_ (3 MPa), 240 °C, 4 h. ^b^ Determined by 2D HSQC NMR. ^c^ Determined by comparison of lignin degradation products with authentic samples on GC. ^d^ **G1**, 4-propylguaiacol; **G2**, 4-*n*-propanol guaiacol; **S1**, 4-propylsyringol; **S2**, 4-*n*-propanol syringol; **3**, methyl 3-(4-hydroxyphenyl)propionate; **4**, methyl 3-(4-hydroxy-3-methoxyphenyl)propionate. ^e^ The mole ratios were determined by comparison of the CEL hydrogenolysis products with authentic samples on GC.

## Data Availability

All data generated or analyzed during this study are included in the article and its information files.

## Supplementary Materials

The supporting information can be downloaded at: https://www.mdpi.com/article/10.3390/ijms241210304/s1.
