# Supplementary material for: Unraveling the Lignin Structural Variation in Different Bamboo Species
Source: Int J Mol Sci. 2023 Jun 18;24(12):10304. doi: 10.3390/ijms241210304 (PMC10299276; doi:10.3390/ijms241210304)
Supplement: Supplementary file 1 [file ijms-24-10304-s001.zip › ijms-2400346-supplementary.pdf]

**Supporting Information (SI) for**

# Unraveling the Lignin Structural Variation in Different Bamboo Species

Ling-Ping Xiao [1,*], Yi-Hui Lv [1], Yue-Qin Yang [1], Shuang-Lin Zou [1], Zheng-Jun Shi [2], and Run-Cang Sun [1,*]

[1] *Liaoning Key Lab of Lignocellulose Chemistry and BioMaterials, Liaoning Collaborative Innovation Center for Lignocellulosic Biorefinery, College of Light Industry and Chemical Engineering, Dalian Polytechnic University, Dalian 116034, China*

[2] *Key Laboratory for Forest Resources Conservation and Utilization in the Southwest Mountains of China, Ministry of Education, Southwest Forestry University, Kunming 650224, China*

* Corresponding authors.

*E-mail addresses*: lpxiao@dlpu.edu.cn (L.-P. Xiao); rsun3@dlpu.edu.cn (R.-C. Sun).

**This PDF file includes:**

1. General information
2. Supplementary Tables. S1 to S4
3. Supplementary Figures. S1 to S7
4. Supplementary references

## 1. General information

### 1.1 Reagents and feedstocks

Bamboo (*Neosinocalamus affinis, Bambusa lapidea*, *Dendrocalamus brandisii*) was harvested from Yunnan Province, China. Methanol (MeOH), dichloromethane (DCM), and tetrahydrofuran (THF) were purchased from Anergy Chemical (China) and used as received without further purification. The commercial cellulase was kindly provided from Novozymes (China) Biotechnology Co., Ltd. Model samples for catalytic degradation was synthesized independently. Lignin model samples for catalytic degradation were synthesized independently. Dimeric lignin model compounds were prepared following previously reported procedures with modifications [1-3].

### 1.2 Preparation of cellulolytic enzyme lignin

The bamboo raw materials were smashed into sawdust (40 ~ 60 mesh), dried in an oven at 60 °C, and then extracted with ethanol/toluene (1:2, v/v) using a Soxhlet extractor for 10 h. The preground and extracted bamboo samples were then planetary ball milled (Fritsch GmbH, Germany) at 400 rpm for 4 h with zirconium dioxide ($ZrO_2$) vessels containing $ZrO_2$ ball bearings (10 mm × 30). One cycle of the ball-milling condition consists of a 10 min milling and a 10 min cooling cycle. Subsequently, the ball-milled samples were subjected to digestion (72 h × 2) to obtain enzyme lignin samples by cellulose at 50 °C in NaOAc buffer (pH 4.8). After that, the solid residue was obtained after centrifugation (5000 rpm for 5 min), washing three times with deionized water, lyophilization, and finally ladled as CEL.

### 1.3 Preparation of acetylated lignin

Briefly, 20 mg lignin was dissolved in 0.5 mL pyridine. To this, 0.5 mL acetic anhydride was added and this mixture was heated for 4 h at 80 ºC. The solution was then added dropwise to 100 mL ice water. The mixture was centrifuged and the water was decanted. A small amount of water was added to the lignin precipitate to sufficiently wash out the pyridine. After centrifugation and washing for repeated three times, the acetylated lignin was then freeze dried.

### 1.4 Catalyst preparation

The Ru@NC catalyst used in this work was prepared and descripted in detail in our previous paper [4]. The carbon carrier (80 mesh) was activated in 38 wt% nitric acid and reacted for 3 h at 80 °C. The treated carbon was washed with deionized water to neutral pH and dried at 110 °C overnight. $RuCl_3$ (0.2043 g) and urea (200 mg) were then added to 50 mL of ethanol and stirred for 2 h. The solution was then injected into the treated carbon using a syringe. The mixture was stirred and heated at 60 °C for 2 h. The resulting solid was collected by filtration and dried under vacuum at 60 °C. The powder was then transferred to a tube furnace and after calcination at 800 °C for 2 h under $N_2$ flow. Finally, the obtained Ru-NPs immobilized on the powder was named as Ru@NC.

**1.5 Catalytic hydrogenolysis of lignin or lignin model compounds**

Typically, CEL (50 mg) or lignin model compounds (15 mg), Ru@NC (5 mg) and MeOH (10 mL) were charged into an autoclave (50 mL, Parr Instrument Company), which was then flushed with $N_2$ for three times and pressurized with 3 MPa $H_2$ at room temperature. Afterwards, the mixture was stirred at 800 rpm and heated to the desired temperature. After the reaction, the autoclave was cooled and depressurized carefully. The reaction mixture was filtered through a nylon 66 membrane filter (0.22 μm), and the insoluble fraction was washed with DCM. Lignin oily product was obtained after removing DCM under a vacuum condition. An external standard (1,3,5-trimethoxybenzene) was added to the lignin oily solution in DCM.

**1.6 Analytical methods**

Elemental analysis was performed on CHNS analyzer (Vario EL III, Element, Germany). Before the test, all the samples were dried at 60 °C in vacuum. Fourier Transform Infrared Spectroscopy (FT-IR) spectra of lignin fractions were performed on a FT-IR spectrophotometer (PerkinElmer, USA). Dried preparations using KBr pellet containing 1% finely ground samples and their spectra were recorded in the range from 4000 to 500 $cm^{-1}$.

Thermogravimetric (TG) and differential thermogravimetric (DTG) analysis of the fractions were investigated by a simultaneous thermal analyzer (TGA-Q500, TA Instruments). The device was heated from room temperature to 700 °C at a heating rate

of 10 °C min$^{-1}$. About 8 ~ 10 mg of samples were weighed each time for thermal stability test under a nitrogen atmosphere of 30 mL min$^{-1}$.

The structural carbohydrates and lignin as well as ash in the dewaxed bamboo sawdust were determined according to the standard analytical procedures (NREL/TP-510-42618 and NREL/TP-510-42622) [5,6]. Briefly, 3 ml 72% $H_2SO_4$ was added to 300 mg dewaxed sawdust and samples were incubated at 30 °C for 1 h. Then 84 ml deionized water was added followed by an incubation for 1 h at 121 °C in an autoclave. The solid residue was dried at 110 °C and weighed as the mass of Klason lignin (AIL). The acid soluble lignin (ASL) in the filtrate was analyzed using a UV-Vis spectrophotometer by recording the absorbance of the soluble fraction at 205 nm. Finally, monosaccharides were determined by high performance anion exchange chromatography with pulsed amperometric detection (HPAEC-PAD). The determination of the ash content in the different bamboo samples was performed uses combustion in a furnace at 600 °C.

Gas chromatography-mass spectrometry (GC-MS) and GC analysis were performed to qualitative and quantitative analysis the aromatic monomers, respectively, as described previously [1,3,7,8]. Briefly, GC-MC analyses of the lignin oily product were carried out on a Shimadzu GCMS-QP2010SE equipped with a HP-5 MS (30 m × 250 μm × 0.25 μm, Agilent) capillary column and an MS detector. GC analyses were conducted with a Shimadzu GC-2010 equipped with a HP-5 column (30 m × 250 μm × 0.25 μm, Agilent) and a flame ionization detector. The monomeric yield obtained from lignins and β-*O*-4′ model compounds were calculated using Equations (1) and (2), respectively:

$$\text{Monomer yield (wt\%)} = \frac{\text{w (monomer)}}{\text{w (initial lignin)}} \times 100\% \quad (1)$$

$$\text{Monomer yield (wt\%)} = \frac{\text{Mole (monomer)}}{\text{Mole (lignin mimics)}} \times 100\% \quad (2)$$

Advanced NMR technologies, including $^{13}C$, $^{31}P$, and 2D HSQC, analysis of lignin or lignin oily products was performed on a Bruker Ascend-400 MHz spectrometer instrument (Bruker, Germany) [1,9]. The molecular weights of lignin and lignin oil were determined by GPC as described previously [1,9].

## 2. Supplementary Tables S1 to S4

**Table S1.** Signal assignment and quantification of functional groups in quantitative $^{13}C$ NMR spectra of the bamboo CELs.

| δ (ppm) | Assignment | $CEL_N$ | $CEL_B$ | $CEL_D$ |
|---|---|---|---|---|
| **Clusters** | | | | |
| 155.0-140.0 | Aromatic C-O | 1.44 | 1.60 | 1.45 |
| 140.0-124.0 | Aromatic C-C | 1.77 | 1.57 | 1.61 |
| 124.0-102.0 | Aromatic C-H | 2.78 | 2.82 | 1.61 |
| 62.0-58.0 | β-*O*-4′ linkages | 0.71 | 0.74 | 0.65 |
| **Individual signals** | | | | |
| 166.7 | $pCA_γ$ | 0.15 | 0.12 | 0.15 |
| 160.1 | $pCA_4$ | 0.15 | 0.10 | 0.12 |
| 155.1-151.1 | $S_{3,5}$ (ether and condensed) | 0.68 | 0.71 | 0.60 |
| 151.0-148.7 | $G_3$ (ether and condensed) | 0.15 | 0.19 | 0.21 |
| 148.7-146.6 | $G_4$ (ether) and $S_{3,5}$ (non-ether) | 0.26 | 0.35 | 0.26 |
| 146.6-144.0 | $G_{3,4}$ (non-ether) | 0.32 | 0.25 | 0.26 |
| 138.1 | $S_4$ (ether) | 0.10 | 0.10 | 0.07 |
| 136.6-133.0 | $S_1/G_1$ | 0.31 | 0.37 | 0.32 |
| 130.5 | $pCA_{2,6}$ | 0.54 | 0.44 | 0.48 |
| 128.1 | $H_{2,6}$ | 0.11 | 0.17 | 0.15 |
| 125.3 | $pCA_1$ | 0.16 | 0.12 | 0.15 |
| 119.5 | $G_6$ | 0.13 | 0.22 | 0.19 |
| 118.0-113.1 | $G_5$, $pCA_β$, $pCA_{3,5}$ and $H_{3,5}$ | 1.19 | 1.10 | 1.12 |
| 113.1-110.0 | $G_2$ | 0.20 | 0.34 | 0.32 |
| 106.5-102.5 | $S_{2,6}$ | 0.78 | 1.01 | 0.85 |
| 102.1 | $T_3$ | 0.12 | 0.08 | 0.09 |
| 97.0 | $T_6$, $T_β$ | 0.02 | 0.01 | 0.03 |
| 88.0-78.0 | $C_β$ in β-*O*-4′, $C_α$ in β-β and β-5 | 0.80 | 0.99 | 0.95 |
| 74.6-70.7 | $C_α$ in β-*O*-4′, $C_γ$ in β-β | 0.81 | 0.87 | 0.78 |
| 64.0-62.3 | $C_γ$ in β-5 and β-1 | 0.31 | 0.44 | 0.32 |
| 61.1-59.2 | $C_γ$ in β-*O*-4′ | 0.45 | 0.75 | 0.51 |
| 56.0 | $-OCH_3$ | 2.40 | 3.07 | 2.37 |

**Table S2.** Assignments of $^{13}C$-$^{1}H$ correlation signals in the HSQC spectra of bamboo CEL and lignin oil obtained from catalytic hydrogenolysis over a Ru@NC catalyst.

| Lables | $\delta_c/\delta_H$ (ppm) | Assignments |
|---|---|---|
| $G1/S1_9$ | 13.8/0.88 | $C_9$-$H_9$ in G1 and G1 products |
| $G1/S1_8$ | 24.0/1.50 | $C_8$-$H_8$ in G1 and G1 products |
| $3/4_7$ | 29.4/2.69 | $C_7$-$H_7$ in 3 and 4 products |
| $G2/S2_7$ | 31.5/2.5 | $C_7$-$H_7$ in G2 and G2 products |
| $G2/S2_8$ | 34.5/1.67 | $C_8$-$H_8$ in G2 and G2 products |
| $3/4_8$ | 35.0/2.54 | $C_8$-$H_8$ in 3 and 4 products |
| $G1/S1_7$ | 37.0/2.40 | $C_7$-$H_7$ in G1 and G1 products |
| CO(OMe) | 51.2/3.52 | C-H in ester groups |
| $C_\beta$ | 53.6/3.05 | $C_\beta$-$H_\beta$ in β-β resinol substructures (C) |
| $-OCH_3$ | 55.7/3.69 | C-H in methoxyls |
| $E_{\alpha(OMe)}$ | 57.5/3.20 | Cα-Hα in γ-methoxylated β-O-4′ substructures (E) |
| $A_\gamma$ | 59.6/3.51 | $C_\gamma$-$H_\gamma$ in γ-hydroxylated β-*O*-4′ substructures (A) |
| $A'_\gamma$ | 63.5/4.21 | $C_\gamma$-$H_\gamma$ in γ-acylated β-*O*-4′ substructures (A′) |
| $G2/S2_9$ | 62.0/3.25 | $C_9$-$H_9$ in G2 and G2 products |
| $B_\gamma$ | 62.8/3.80 | $C_\gamma$-$H_\gamma$ in pheylcoumaran substructures (B) |
| $A_\alpha/A_\alpha'$ | 71.8/4.80 | $C_\alpha$-$H_\alpha$ in β-*O*-4′ units (A and A′) |
| $C_\gamma$ | 71.0/3.81-4.13 | $C_\gamma$-$H_\gamma$ in β-β resinol substructures (C) |
| $B_\alpha$ | 86.9/5.42 | $C_\alpha$-$H_\alpha$ in pheylcoumaran substructures (B) |
| $C_\alpha$ | 85.0/4.61 | $C_\alpha$-$H_\alpha$ in β-β resinol substructures (C) |
| $D'_{(\alpha)}$ | 80.8/3.85 | $C'_\alpha$-$H'_\alpha$ in dibenzodioxocin substructures (D′) |
| $A_{\beta(G/H)}$ | 83.5/4.32 | $C_\beta$-$H_\beta$ in β-*O*-4′ substructures linked (A) to G/H units |
| $A_{\beta(s)}$ | 85.9/4.15, 86.3/4.26 | $C_\beta$-$H_\beta$ in β-*O*-4′ substructures linked (A) to a S unit |
| $T_8$ | 94.3/6.62 | $C_8$-$H_8$ in tricin (T) |
| $T_6$ | 98.9/6.15 | $C_6$-$H_6$ in tricin (T) |
| $T_{2'/6'}$ | 103.9/7.30 | $C_{2',6'}$-$H_{2',6'}$ in tricin (T) |
| $S_{2/6}$ | 103.9/6.58 | $C_{2,6}$-$H_{2,6}$ in syringyl units (S) |
| $S'_{2/6}$ | 106.1/7.29 | $C_{2',6'}$-$H_{2',6'}$ in syringyl units (S′) |
| $FA_2$ | 110.9/7.25 | $C_2$-$H_2$ in ferulate (FA) |
| $FA_\beta$ | 115.3/6.29 | $C_8$-$H_8$ in ferulate (FA) |
| $G_2$ | 110.9/6.93 | $C_2$-$H_2$ in guaiacyl (G) |
| $G_5$ | 114.4/6.69 | $C_5$-$H_5$ in guaiacyl (G) |
| $pCA_\beta$ | 114.5/6.15 | $C_\beta$-$H_\beta$ in *p*-coumarate (*p*CA) |
| $pCA_{3/5}$ | 113.8/6.20 | $C_2$-$H_2$ and $C_{3,5}$-$H_{3,5}$ in *p*-coumarate (*p*CA) |
| $G_6$ | 118.9/6.80 | $C_6$-$H_6$ in guaiacyl (G) |
| $H_{2/6}$ | 127.8/7.28 | $C_{2,6}$-$H_{2,6}$ in H units (H) |
| $pCA_{2/6}$ | 130.0/7.35 | $C_2$-$H_2$ and $C_{2,6}$-$H_{2,6}$ in *p*-coumarate (*p*CA) |
| $pCA_\alpha$ | 144.4/7.35 | $C_\alpha$-$H_\alpha$ in *p*-coumarate (*p*CA) |

**Table S3.** Hydroxyl groups (mmol/g) contents of the bamboo CELs as determined by $^{31}P$ NMR.

| | Lignin samples | | |
|---|---|---|---|
| | $CEL_N$ | $CEL_B$ | $CEL_D$ |
| Aliphatic-OH | 3.03 | 3.81 | 3.32 |
| Syringyl-OH | 0.30 | 0.33 | 0.32 |
| Guaiacyl-OH (condensed) | 0.02 | 0.05 | ND |
| Guaiacyl-OH (non-condensed) | 0.24 | 0.48 | 0.41 |
| Total phenolic OH | 0.56 | 0.69 | 0.64 |
| COOH | ND | 0.15 | 0.07 |

**Table S4.** Chemical characteristics and product yields of the bamboo, CELs and lignin oil obtained from the catalytic hydrogenolysis of CEL over a Ru@NC catalyst.

| Sample | Elemental composition (%) | | | | | | Atomic ratio | | HHV (MJ/Kg)[a] |
|---|---|---|---|---|---|---|---|---|---|
| | C | H | O | N | S | Ash | O/C | H/C | |
| $Bamboo_N$ | 48.46 | 5.48 | 36.72 | 6.20 | 0.13 | 3.01 | 0.57 | 1.36 | 17.66 |
| $Bamboo_B$ | 48.77 | 5.51 | 37.79 | 6.09 | 0.11 | 1.73 | 0.58 | 1.26 | 17.62 |
| $Bamboo_D$ | 48.50 | 5.63 | 39.10 | 4.91 | 0.10 | 1.70 | 0.61 | 1.39 | 17.46 |
| $CEL_N$ | 59.11 | 5.34 | 27.78 | 7.58 | 0.19 | / | 0.35 | 1.08 | 22.65 |
| $CEL_B$ | 61.03 | 5.27 | 26.90 | 6.65 | 0.15 | / | 0.33 | 1.04 | 23.39 |
| $CEL_D$ | 60.89 | 5.23 | 26.73 | 6.99 | 0.16 | / | 0.33 | 1.03 | 23.28 |
| Lignin $Oil_N$ | 61.79 | 6.53 | 23.30 | 8.31 | 0.07 | / | 0.28 | 1.27 | 26.05 |
| Lignin $Oil_B$ | 61.90 | 6.58 | 25.12 | 6.31 | 0.09 | / | 0.30 | 1.28 | 25.83 |
| Lignin $Oil_D$ | 64.28 | 6.48 | 22.36 | 6.74 | 0.14 | / | 0.26 | 1.21 | 26.99 |

[a] HHV: higher heating value. Evaluated using the Dulong's formula: HHV = 0.3383 × C + 1.422 × (H-O/8).

## 3. Supplementary Figures S1 to S7

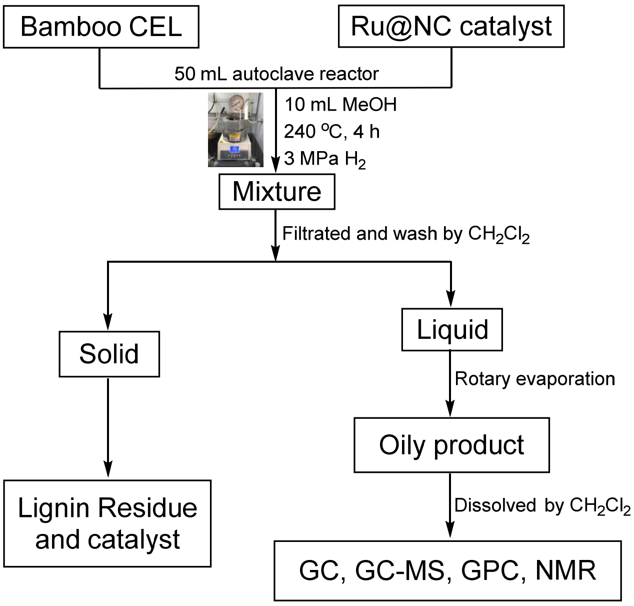


**Figure S1.** Schematic representation of catalytic hydrogenolysis of bamboo CELs over a Ru@NC catalyst.

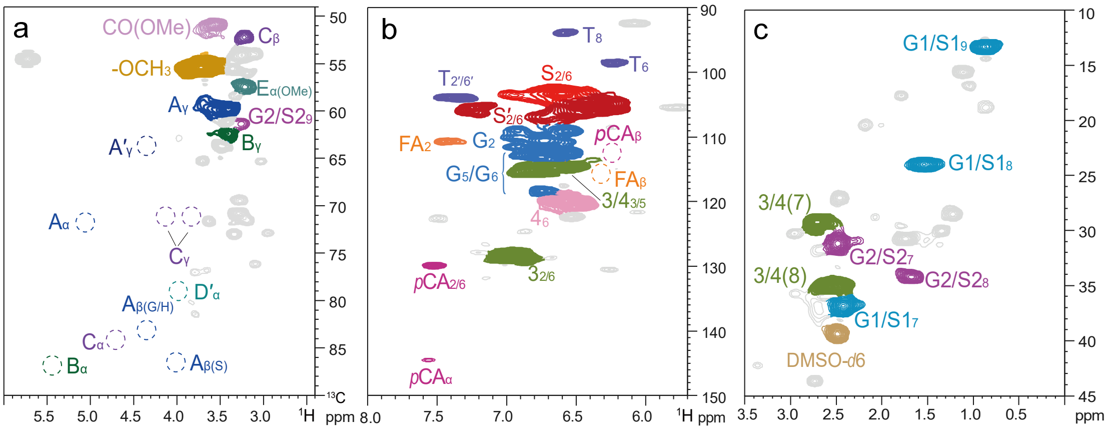


**Figure S2.** 2D HSQC NMR spectra of lignin oily product (**a**,**b**,**c**) obtained from CEL$_B$ after catalytic hydrogenolysis over a Ru@NC catalyst.

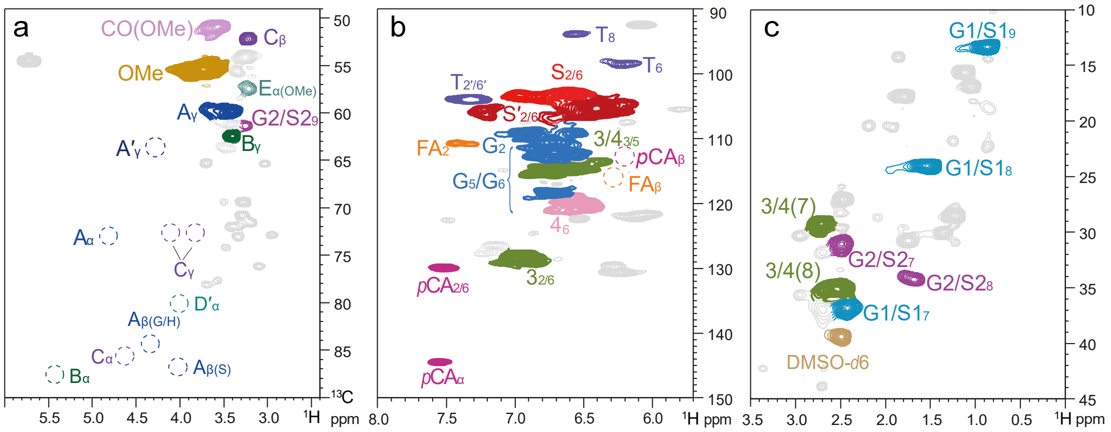


**Figure S3.** 2D HSQC NMR spectra of lignin oily product (**a**,**b**,**c**) obtained from CEL<sub>D</sub> after catalytic hydrogenolysis over a Ru@NC catalyst.

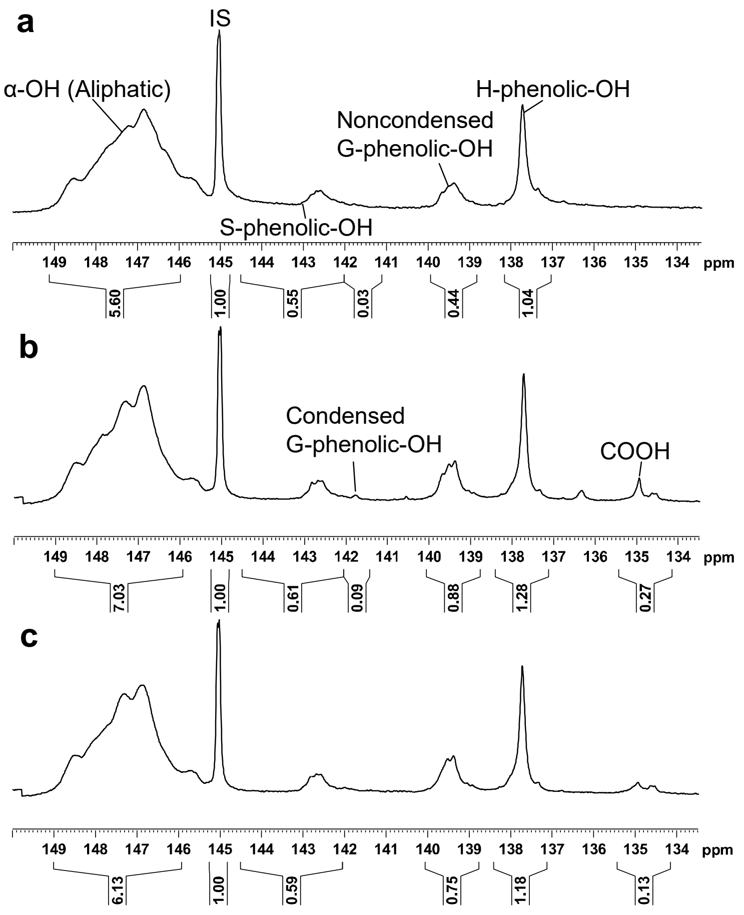


**Figure S4.** $^{31}$P NMR spectra of the (**a**) CEL$_N$, (**b**) CEL$_B$, and (**c**) CEL$_D$.

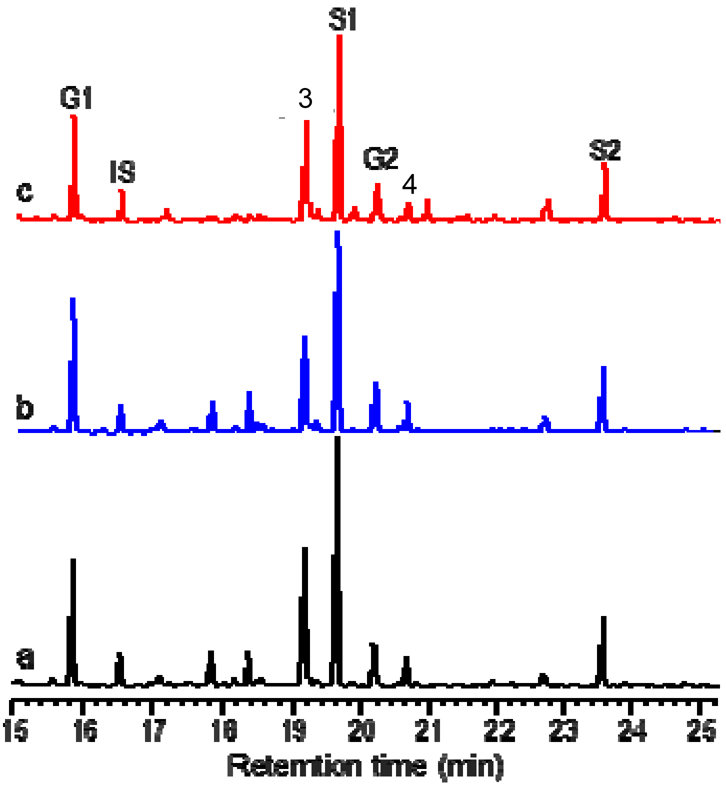


**Figure S5.** Gas chromatogram and peak identification of lignin oil obtained from (a) $CEL_N$, (b) $CEL_B$, and (c) $CEL_D$ after catalytic hydrogenolysis over a Ru@NC catalyst.

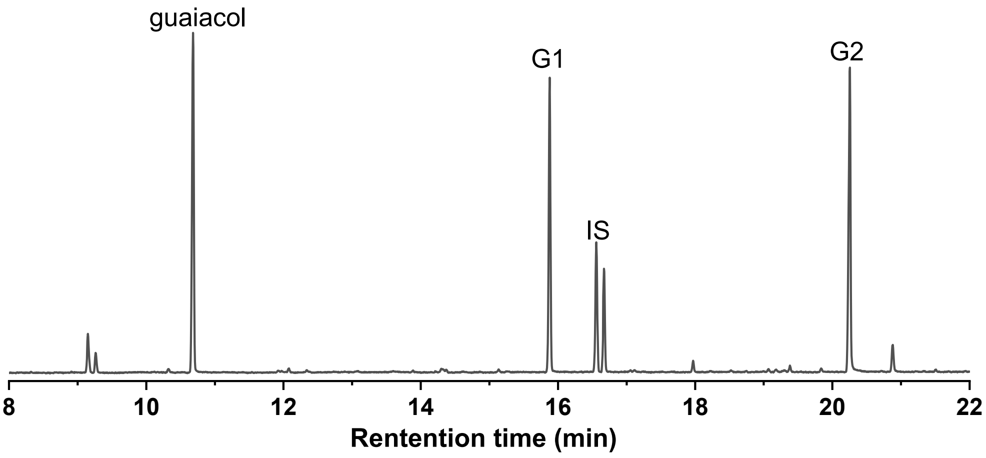


**Figure S6.** Gas chromatogram of lignin oily products obtained from the catalytic hydrogenolysis of the phenolic β-*O*-4′ model compound **1** over a Ru@NC catalyst. IS represents internal standard (1,3,5-trimethoxybenzene).

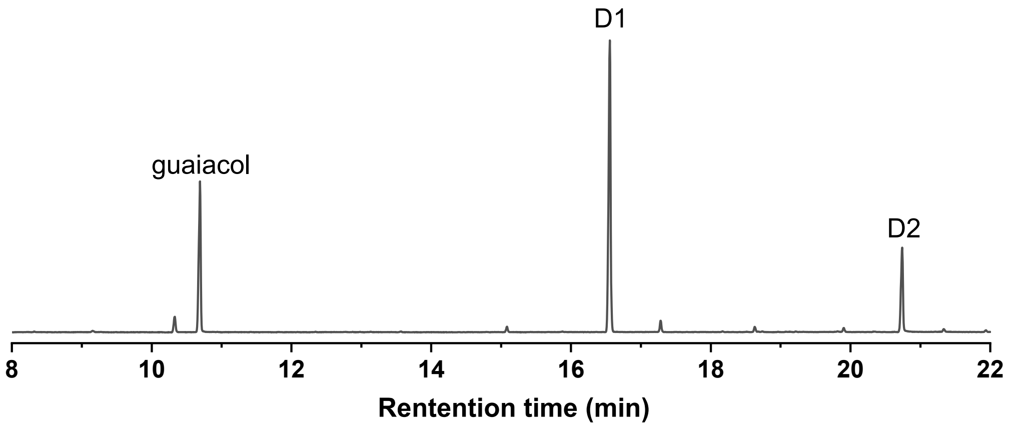


**Figure S7.** Gas chromatogram of lignin oily products obtained from the catalytic hydrogenolysis of the nonphenolic β-*O*-4′ model compound **2** over a Ru@NC catalyst.

## 4. Supplementary References


[1] Wang, Q.; Xiao, L.-P.; Lv, Y.-H.; Yin, W.-Z.; Hou, C.-J.; Sun, R.-C. Metal–Organic-Framework-Derived Copper Catalysts for the Hydrogenolysis of Lignin into Monomeric Phenols. *ACS Catal.* **2022**, *12*, 11899-11909, doi:10.1021/acscatal.2c02955.

[2] Liu, Z.; Li, H.; Gao, X.; Guo, X.; Wang, S.; Fang, Y.; Song, G. Rational highly dispersed ruthenium for reductive catalytic fractionation of lignocellulose. *Nat. Commun.* **2022**, *13*, 4716, doi:10.1038/s41467-022-32451-5.

[3] Xiao, L.-P.; Wang, S.; Li, H.; Li, Z.; Shi, Z.-J.; Xiao, L.; Sun, R.-C.; Fang, Y.; Song, G. Catalytic hydrogenolysis of lignins into phenolic compounds over carbon nanotube supported molybdenum oxide. *ACS Catal.* **2017**, *7*, 7535-7542, doi:10.1021/acscatal.7b02563.

[4] Yang, Y.-Q.; Xiao, L.-P.; Xiao, W.-Z.; Li, X.-Y.; Wang, Q.; Sun, R.-C. Nitrogen-doped carbon anchored ruthenium nanoparticles for biofuel upgrade. *Fuel* **2022**, *314*, 123100. doi:10.1016/j.fuel.2021.123100.

[5] Sluiter, A.; Hames, B.; Ruiz, R.; Scarlata, C.; Sluiter, J.; Templeton, D.; Crocker, D. Determination of structural carbohydrates and lignin in biomass. *Laboratory Analytical Procedure (NREL/TP-510-42618)*, *NREL: Golden*, CO, USA, **2008**.

[6] Sluiter, A.; Ruiz, R.; Scarlata, C.; Sluiter, J.; Templeton, D. Determination of Extractives in Biomass, *Laboratory Analytical Procedure (NREL TP-510-42619)*, *NREL: Golden*, CO, USA, **2008**.

[7] Su, S.; Xiao, L.-P.; Chen, X.; Wang, S.; Chen, X.-H.; Guo, Y.; Zhai, S.-R. Lignin-First Depolymerization of Lignocellulose into Monophenols over Carbon Nanotube-Supported Ruthenium: Impact of Lignin Sources. *ChemSusChem* **2022**, *15*, e202200365, doi: 10.1002/cssc.202200365.

[8] Wang, S.; Li, W.-X.; Yang, Y.-Q.; Chen, X.; Ma, J.; Chen, C.; Xiao, L.-P.; Sun, R.-C. Unlocking structure-reactivity relationships for catalytic hdrogenolysis of lignin into phenolic monomers. *ChemSusChem* **2020**, *13*, 4548-4556, doi:10.1002/cssc.202000785.

[9] Li, W.-X.; Xiao, W.-Z.; Yang, Y.-Q.; Wang, Q.; Chen, X.; Xiao, L.-P.; Sun, R.-C. Insights into bamboo delignification with acidic deep eutectic solvents pretreatment for enhanced lignin fractionation and valorization. *Ind. Crops Prod.* **2021**, *170*, 113692, doi:10.1016/j.indcrop.2021.113692.
